# Traditional Chinese Medicine Strategy for Patients with Tourette Syndrome Based on Clinical Efficacy and Safety: A Meta-Analysis of 47 Randomized Controlled Trials

**DOI:** 10.1155/2021/6630598

**Published:** 2021-03-10

**Authors:** Na Wang, Dong-dong Qin, Yu-huan Xie, Xin-chen Wu, Ding-yue Wang, Hang-Yang, Xiao-xuan Li, Lei Xiong, Jing-hong Liang

**Affiliations:** ^1^School of Basic Medical Science, Shanghai University of Traditional Chinese Medicine, Shanghai 201203, China; ^2^School of Basic Medical Science, Yunnan University of Chinese Medicine, Yunnan Kunming 650500, China; ^3^First Affiliated Hospital, Yunnan University of Chinese Medicine, Yunnan Kunming 650500, China; ^4^Department of Maternal and Child Health, School of Public Health, Sun Yat-sen University, Guangzhou 510080, China

## Abstract

**Objective:**

Although increasing evidence reveals the efficacy of traditional Chinese medicine (TCM) and its safety on Tourette Syndrome (TS) patients, whether TCM is indeed improving TS remains unclear. The purpose of the current study is to perform a meta-analysis to evaluate the efficacy and safety of TCM on treating TS patients.

**Method:**

An elaborate search strategy was conducted based on several databases including Medline, Embase, Cochrane, Web of Science, CINAHL, CBM, VIP, CNKI, and Wanfang Data in order to identify the relevant randomized controlled trials (RCTs) from their inception to as late as May 1st, 2020. General information and data needing analysis were extracted simultaneously for the necessity of various analyses such as descriptive analysis and metaquantitative analysis.

**Results:**

Forty-seven trials with 5437 TS patients in total were eventually included according to our criteria. All trials were conducted in China, and the publication years ranged from 2004 to 2017. In terms of clinical efficacy, clinical symptoms of patients with TCM were more likely to be improved compared with the control group (odds ratio, OR = −1.29, 95% confidence interval, CI: -2.54 to -0.06, *I*^2^ = 0.00%). As to the outcome of recurrence rate, the pooled results revealed that the TCM group was more inclined to stabilize the recurrence (OR = 0.44, 95% CI: 0.24 to 0.78, *I*^2^ = 0.00%). Similar results were observed in adverse reaction (OR = 0.32, 95% CI: 0.24 to 0.43, *I*^2^ = 32.90%).

**Conclusion:**

The results of our study recommend applying TCM to treat TS patients for better efficacy and safety. Results need to be interpreted cautiously due to certain limitations in our study.

## 1. Introduction

Tourette Syndrome (TS), a type of neurodevelopment disturbance, is commonly characterized by the occurrence of sudden, brief, intermittent movements or vocalization, with a high incidence rate among young children (three to eight cases out of 1000) [1] and over 1% globally [2]. Symptoms of TS patients are frequently accompanied by cooccurring disorders, such as attention-deficit/hyperactivity disorder (ADHD), anxiety disorder, obsessive-compulsive disorder, and schizophrenia [3, 4]. The symptoms and complications of TS will trigger a series of consequences for both the patients and their families, even the society sometimes, with TS patients' life quality harmed, financial burden increased, and social resources consumed [5, 6].

In practice, guanfacine and clonidine are recommended as the first choice in the treatment of TS patients, along with atypical drugs functioning as dopamine receptor antagonists, namely, antipsychotics such as tiapride and haloperidol, whose efficacy has been proved in numerous clinical trials [7, 8]. However, previous researches also indicated that the mentioned drugs may bring serious adverse reactions (ARs) to patients [9, 10]. Therefore, in the absence of a universally accepted and reasonable treatment and with new clinical trials to seek possible drug candidates evolving frequently, there is an urgent need for clinical researchers to figure out a reliable treatment to solve this disorder [11].

Currently, complementary and alternative medicine is arresting more attention for their relatively higher efficacy and safety than traditional first-tier drugs, and some studies even point out that 64% TS patients have received this kind of relatively effective clinical approach for now [12]. As a branch of alternative medicine and ethnopharmacology that dates back to ancient China, traditional Chinese medicine (TCM) has been widely supported by clinical trials for its efficacy and lower rate of recurrence and undesirable side effects [13]. Developed based on traditional and modern medicine theories, TCM, or TCM-assisted treatments, with obvious advantages of high bioavailability and quick effect, is widely used for treating various clinical diseases and, to some extent, effective in relieving the clinical symptoms, such as primary nephrotic syndrome, poststroke depression, or community-acquired pneumonia [14–16]. Although several trials have examined the effectiveness of TCM in treating TS, there have been few studies to combine the available evidence of trials to explore the potential relationship between TCM and TS in terms of efficacy and safety. Two groups of comparisons were conducted. The first compared the effectiveness of TCM with placebo and Western medicine (WM), the second the effectiveness of combined TCMs with WM. The study, however, only quantitatively analyzed the efficacy of TCM based on limited outcomes and studies [17]. Furthermore, the quality of studies included and insufficient analysis from previous meta-analyses were prone to threaten the conclusion. Accumulating evidence regarding the efficacy and safety of TCM on TS was fragmented and inconsistent. Therefore, we formulate a hypothesis to identify the efficacy and safety of TCM in treating TS patients by maximizing, summarizing, and analyzing the available evidence.

## 2. Method

Our study was performed as instructed by the Preferred Reporting Items for Systematic Reviews and Meta-Analyses (PRISMA) statement [18], the Cochrane Collaboration Handbook recommendations [19]. No ethical approval or patient consent was required in that all analyses were conducted based on previously published studies.

With no language or publication time restrictions, a comprehensive literature search was carried out to identify the relevant randomized controlled trials (RCTs) which investigated the efficacy of traditional Chinese medicine on TS in the following electronic databases: Medline (via PubMed), Embase, Cochrane, Web of Science, CINAHL, Chinese National Knowledge Infrastructure, Wanfang Data, Chinese Scientific Journal database, Chinese Biomedical Literature Database, Chinese Clinical Trial Registry, and clinical trials (http://www.clinicaltrials.gov) from their inception to May 1^st^, 2020.

Through the medical subject headings (MeSH) incorporated with free text terms by using the Boolean logical operators, an exhaustive search was performed, with the following terms: “Tourette Syndrome”, “Traditional Chinese medicine”, “Drugs, Chinese herbal”, “Randomized controlled trial” taken into account. Moreover, we conducted a series of recursive searches as complementary retrieval from top journals (top journals in China: China Journal of Chinese Materia Medica, Journal of Traditional Chinese Medicine, China Journal of Traditional Chinese Medicine and Pharmacy, and China Pharmacy; top international journals: Science China Life Sciences, Frontiers in Pharmacology, Frontiers in Microbiology, International Journal of Biological Macromolecules, and Pharmacological Research), famous publishers, major international conference proceedings, and grey literature ((theses of doctors' and masters' noncommercial bibliography, technical documents (including government reports)) to minimize the loss of omission of suitable articles that meet our inclusion criterion. Bibliography from included studies and similar meta-analyses and systematic reviews were additionally screened for potentially eligible studies. Details of all databases of search strategies were shown in supplement search strategies.

A check was indispensable for the integrity and veracity of studies. All records from the initial search were imported into EndNote X9 (Thomson ISI Research Soft, Philadelphia, Pennsylvania, USA), managing and confirming the above information which was performed concurrently by two independent authors. Discrepancies during this progress were settled by discussion or judged by the third author.

### 2.1. Eligibility Criteria and Selection Process

Studies were included with the PICOS criteria as follows:

#### 2.1.1. Population

Participants were of any age diagnosed with TS (neurodevelopment disturbance commonly characterized by the occurrence of sudden, brief, intermittent movements or vocalization) by proper medical diagnosis criteria, such as the Diagnostic and Statistical Manual, fourth edition, or the text revision of the fourth edition (DSM-V/IV) [20], the Chinese Classification of Mental Disorders (CCMD), whether the patients were diagnosed according to CCMD-2R or CCMD-3.

#### 2.1.2. Interventions

Acceptable treatments included any structured and conceptualized traditional Chinese medicine (TCM), such as Ningdong granule, Qufeng Zhidong recipe. Any CTM in combinatorial or multicomponent was excluded.

#### 2.1.3. Comparators

Studies were included if their comparison groups were set as any placebo such as active Western medicine, nutrient, while the studies were excluded when their control groups (CGs) were conducted in any combination with any placebo.

#### 2.1.4. Outcomes

As an indication of reporting the response rate that both occurred in the treatment and control group, the primary outcome was clinical efficacy (CE) which was measured by a variety of rating clinical instruments. Secondary outcomes were other clinical indications, such as Yale Global Tic Severity Scale (YGTSS) [21], adverse reaction (AR), and TCM Syndrome Score Scale (TCMSSS), a widely used clinical tool designed in a questionnaire and monitored by the Xiangya Hospital.

#### 2.1.5. Study Design

Only the parallel-group RCTs that have been published with no language restrictions were involved.

As defined in our included and excluded criteria, duplications were removed first. Meanwhile, two authors independently selected the studies by screening their titles and abstracts. Subsequently, full-text reviewing was performed in an effort to retrieve the potentially eligible studies. Inconsistencies that emerged in this part were avoided.

### 2.2. Data Collection and Quality Assessment

Based on the Cochrane Consumers and Communication Review Group's data extraction template [19], a rigorous process of data inspection was performed to extract the key data of included studies by two authors independently. The following relevant items were collected on the basis of the preelaborated measurement: major author responsible for the study, publication year, total sample size, therapeutic course, various outcomes, area of study, etc.

Two investigators independently applied the Cochrane Risk of Bias tool (ROB) in the evaluation of the quality of each included study [19], which consisted of seven items (random sequence generation, allocation concealment, blinding of participants and personnel, blinding of outcome assessment, incomplete outcome data, selective reporting, and other biases), and each of the items was rated as unknown, low, and high risk of bias, respectively.

### 2.3. Statistical Analyses

Based on the Cochrane Collaboration Handbook recommendations [22], a conventional pairwise meta-analysis of crossed trials was conducted for each comparison. Firstly, in terms of statistical heterogeneity, *I*^2^ statistics, whose values were 25%, 50%, and 75%, respectively, indicating mild, moderate, and high heterogeneity, were provided to determine whether there was a substantial heterogeneity produced [23]. Secondly, for the outcomes presented as numerical variables, we either extracted their mean difference and standard deviation of the change from baseline or transformed them into a standard format to ensure that our analyses were smoothly performed. Moreover, in the presence of effect sizes referring to continuous outcome, standard mean differences (SMDs) were calculated for each comparison using group (relevant) means and standard deviations (SD) from individual studies. For dichotomous variables, the odds ratio (OR) was used to compute the pooled effect sizes (ESs) for each study with the random-effects model [24], whose 95% of confidence interval (CI) were calculated along with the two combined ES above as a measurement of estimated uncertainty. Thirdly, if the number of included studies in relevant outcomes is over ten, the comparison-adjusted funnel plot based on it will be drawn to detect the presence of any prominent types of potential bias through intuitive vision, such as publication bias, selective reporting, or other biases. As a quantitative complement for the funnel plot, the Egger test was additionally conducted to observe whether the *P* value was less than 0.05 [25]. At last, based on various interesting variables, planned random-effects subgroup analyses were conducted to ensure the robustness of the summarized ES and all of them were judged as preestablished concomitant variables. A sequence of areas was taken into consideration in our model shown as follows: course of disease (average course ≤ 3 years vs. average course > 3 years); condition of disease (mild vs. moderate-severe); publication year (year ≤ 2010 vs. year > 2010); therapeutic course (duration weeks ≤ 12 vs. duration weeks > 12); diagnostic standards (CCMD vs. DSM vs. other criteria); total sample size (sample size ≤ 100 vs. sample size > 100); region (economically backward vs. developed economy); risk of bias group (high quality vs. low and unclear quality group). Furthermore, metaregression based on the random effects was carried out to explore the sources of existing heterogeneity by adjusting the potentially confounding factors if necessary [23]. The above battery of analyses was performed in STATA, version 15.0 (StataCorp, College Station, TX).

## 3. Results

### 3.1. Literature Selection and Characteristics of Included Studies

**Figure 1:**
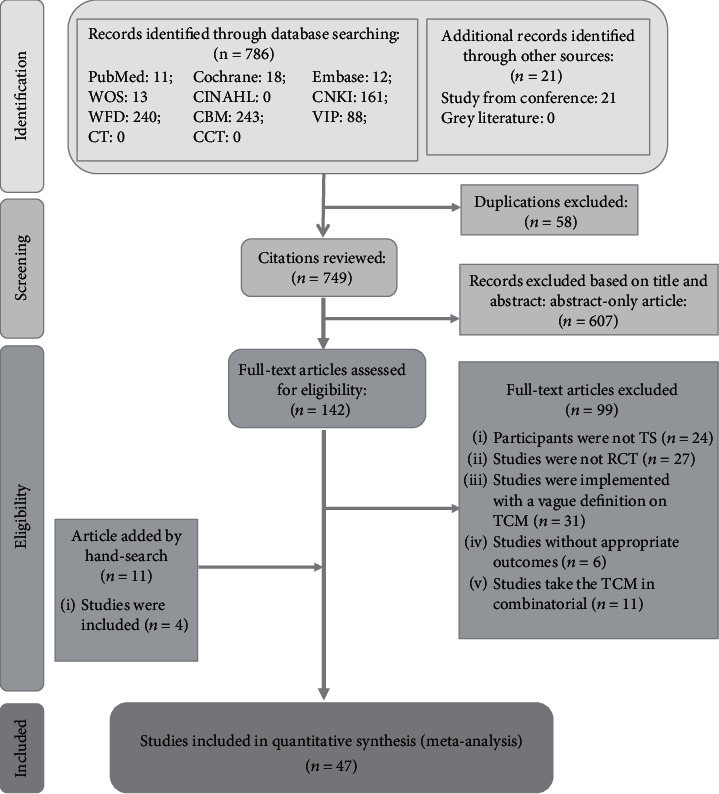
Literature review flowchart. CBM = Chinese Biomedical Literature Database; CCT = Chinese Clinical Trial Registry; CNKI = China National Knowledge Infrastructure database; CT = clinical trials; RCT = randomized controlled trial; TS = Tourette Syndrome; VIP = Chinese Scientific Journal database; WFD = Wanfang database; WOS = Web of Science.

The initial target databases search yielded 807 records while 21 were found by extra manual search, among which 58 articles were removed due to duplication. As a result, the number reduced to 142 according to the title and abstract screen results. After the full-text review, 99 studies were excluded, whose contents were irrelevant as follows. Patients of 24 studies were not diagnosed with TS, and 27 studies were not launched as RCT. Meanwhile, 31 studies did not have an explicit definition on TCM and 6 studies did not report the related outcomes. Furthermore, 11 studies took the TCM in combinatorial. At last, we retrieved 11 studies by hand search and 4 were eligible to be included. In that case, 47 unique trials [26–72] in total were finally included in our study. Details of the selection process are displayed in Figure 1.

All 47 trials were conducted in China with publication years ranging from 2004 to 2017. Altogether, 3231 participants were randomized to the TCM group while the control group comprised 2206 participants with an overall median age of 8.17 years old. The proportion of the male patients (*N* = 3549) was significantly higher than female patients (*N* = 1228) (two studies were not reported). Participants recruited and recorded in all trials met at least one standard diagnostic criterion, such as DSM-IV (*N* = 30), CCMD (*N* = 10), or other clinical diagnostic instruments (*N* = 7). The disease course of patients with TS ranged from 1 to 9 years old, their therapeutic course 3 to 24 weeks. Demographic characteristics of the 47 trials are described in Table 1.

### 3.2. Quality of Included Studies

A relatively low or unclear risk of bias was obtained in 40 of the included trials while 7 studies were categorized as high risk of bias [36, 37, 39, 41, 62, 70, 71]. Sufficient generation of random sequence was observed in all 47 trials whereas few of them had given their allocation concealment. Only 8 trials [38, 47, 53, 59–61, 66, 69] have mentioned adequate blinding of participants and personnel while the remaining studies were unclear. The item for evaluating the outcome was shown a relative completeness, except these trials [36, 37, 39, 70, 71]. Three trials were judged as high risk of bias based on other bias items [37, 41, 62]. Overall and individual quality was detailed in Supplement Figure 1 and Supplement Figure 2.

### 3.3. Primary Outcome

#### 3.3.1. Clinical Efficacy

All 47 trials investigated the relationship of response rate between TCM and CG, and our results showed that TCM was more likely to take effect than the CG in clinical efficacy with a statistical significance OR of 1.15 (OR = 1.15, 95% CI: 1.06 to 1.26, *I*^2^ = 0.00%) (shown in Table 2). The funnel plot hint of no publication bias was observed (Supplement Figure 3), and equal indication was found in the quantitative egger test (*P* value = 0.007) (shown in Supplement Figure 4).

### 3.4. Secondary Outcome

#### 3.4.1. Yale Global Tic Severity Scale

There were 26 studies concentrated on YGTSS total scores. Results presented that patients who received TCM were notably improving their YGTSS total scores when compared with the CG (SMD = −0.21, 95% CI: -0.29 to -0.14, *I*^2^ = 12.30%) (shown in Table 2).

In terms of the YGTSS motor tic scores, an item of YGTSS, 9 studies targeted at it and the pooled results reported remarkable improvement of the TCM group compared with the CG ((OR = 1.26, 95% CI: 1.002 to 1.58, *I*^2^ = 0.00%) and (SMD = −0.37, 95% CI: -0.55 to -0.19, *I*^2^ = 41.50%)). Likewise, results from 7 studies revealed that patients with TS in the TCM group got a significant improvement in YGTSS vocal tic scores (SMD = −0.23, 95% CI: -0.35 to -0.10, *I*^2^ = 0.00%), but a nonsignificant improved performance was detected while the ES was accounted in a dichotomous variable (OR = 1.17, 95% CI: 0.92 to 1.49, *I*^2^ = 0.00%) (shown in Table 2).

#### 3.4.2. Traditional Chinese Medicine Syndrome Score Scale

14 and 6 studies recorded the endpoints of TCMSSS with a dichotomous variable and numerical variable, respectively. Among included studies with different variable types, meta-analysis results showed that compared with the CG, the TCM group had a conspicuous improvement ((OR = 1.20, 95% CI: 1.04 to 1.37, *I*^2^ = 0.00%) and (SMD = −0.69, 95% CI: -1.17 to -0.21, *I*^2^ = 80.50%)) (shown in Table 2).

#### 3.4.3. Recurrence Rate and Adverse Reaction

Six studies used recurrence rate as one of their endpoints. Furthermore, there were 29 (studies) collecting adverse reaction as their endpoints. When it comes to recurrence rate, the TCM group marked a higher likelihood to obviously contain the recurrence (OR = 0.44, 95% CI: 0.24 to 0.78, *I*^2^ = 0.00%). As for adverse reaction, combined data regarding the TCM group was prominent in reducing the adverse effect (OR = 0.32, 95% CI: 0.24 to 0.43, *I*^2^ = 32.90%) (shown in Table 2).

*I* ^2^ values reflected that there existed mild magnitude of heterogeneity across the included studies based on the outcome of YGTSS motor tic scores and adverse reaction, and severe heterogeneity in TCMSSS (shown in Table 2).

### 3.5. Subgroup Analyses

The prespecified subgroup analyses are eventually shown in Table 2, which was divided into 8 categories. The overwhelming majority of subgroup analyses yielded consistent results, i.e., items under the subgroup were of statistical significance (illness course group, (average course ≤ 3 years, OR = 1.14, 95% CI: 0.99 to 1.30) vs. (average course > 3 years, OR = 1.15, CI:0.93 to 1.42); condition of the disease group, (mild, OR = 1.06, 95% CI: 0.71 to 1.61) vs. (moderate-severe, OR = 1.09, 95% CI: 0.80 to 1.48); diagnostic standards, (CCMD-II/III, OR = 1.18, 95% CI: 1.00 to 1.39) vs. (DSM-IV, OR = 1.15, 95% CI:1.09 to 1.29); risk of bias group, (high risk of bias, OR = 1.15, 95% CI: 1.06 to 1.26) vs. (low and unclear risk of bias, OR = 1.16, 95% CI:1.06 to 1.28)); however, when the publication years were taken into consideration, years later than 2010 (OR = 1.18, CI: 1.06 to 1.32) saw a notable improvement compared with the CG while the remaining (cases) whose publication years were no later than 2010 had little improvement by detection (OR = 1.10, CI: 0.96 to 1.27). In terms of the total sample size group, the sample size ≤ 100 (OR = 1.11, CI: 0.98 to 1.26) showed an inconsistency with the sample size > 100 (OR = 1.19, CI: 1.05 to 1.34) that the former experienced a remarkable improvement of TS patients. Finally, the results in regions with low or middle income (OR = 1.18, CI: 1.06 to 1.32) were prominently superior to the CG and regions with high income (OR = 1.11, CI: 0.97 to 1.27).

## 4. Discussion

From the overall results of our study involving 47 RCTs and 5437 patients, compared with the control group, TS patients assigned to the TCM group witnessed a higher possibility of more effective treatment in clinical efficacy (OR = 1.15, 95% CI: 1.06 to 1.26), YGTSS total scores (SMD = −0.21, 95% CI: -0.29 to -0.14), TCMSSS (OR = 1.20, 95% CI: 1.04 to 1.37), recurrence rate (OR = 0.44, 95% CI: 0.24 to 0.78), and adverse reaction (OR = 0.32, 95% CI: 0.24 to 0.43), while it remained to be seen whether TCM could improve YGTSS vocal tic scores of TS patients (OR = 1.17, 95% CI: 0.92 to 1.49). The summarized findings above suggested that TCM might achieve a relative effectiveness to alleviate the clinical symptoms of TS patients with a low risk of adverse reactions.

A high proportion of 47 included studies set their control group as the Western medicine (WM) of antipsychotics such as haloperidol and tiapride, which have been recommended by The European Society for the Study of Tourette Syndrome (ESSTS) as the medications to treat TS patients [73, 74]. However, pooled results from our study indicated that the intervention group of TCM was apparently more effective than WM in clinical efficacy rate or adverse effect. Chinese medicine categorizes TS into three types, namely, convulsion, muscular twitch, and cramp [44]. Most TS patients would experience symptoms like repetitive respiratory tract infection, nonrhythmic tics, which are caused by “wind invasion” into the lung—a “lung treatment” theory in TCM, accounting for the streptococcal infection, whose common symptoms are fever, stuffy nose, and sore throat [45]. Based on the visceral function of Chinese medicine, in order to improve the symptoms of TS patients, TCM often uses a combination of multiple Chinese herbals as their prescription to alleviate the wind and drive the chill out of the mind to maintain body homoeostasis [44]. Ningdong granule is regarded as a widespread Chinese prescription consisting of eight Chinese herbals with the function of antispasm, antiepilepsy, and sedation. It can further take effect as a nonspecific immune role with various degrees to alleviate the conditions of spasms and convulsions by inhibiting the hemolytic streptococcus and Streptococcus viridans [44]. As the authoritative scale for measuring the clinical effectiveness of TS patients, the total scores of YGTSS meant an obvious reduction in the TCM group which was consistent with the previous studies even in insufficient trials included [17]. Our main findings reinforce the existing evidence that TCM was more inclined to function in the treatment of tic symptom.

Not only was the superiority reflected in its effectiveness but also it was endorsed by a stable adverse effect. TS patients treated with WM (haloperidol, risperidone, etc.) were more prone to suffer from dizziness, drowsiness, and constipation even the extrapyramidal system reaction easily. Nonetheless, the adverse reactions that occurred among patients who received TCM were less frequently observed than those observed in WM [69, 75]. Furthermore, TCM, with its advantages of efficacy in both the short term and the long term, proved itself worth introducing [44]. Apparently, as an essential component of Chinese medicine, acupuncture with its promising effectiveness seems to be as reputed as TCM and some articles indicated that the combination of acupuncture and TCM would optimize the effectiveness brought by the treatment [76, 77].

Based on the primary outcome, several significant differences were observed and introduced as follows. Compared with the studies with publication years no later than 2010 (OR = 1.10, CI: 0.96 to 1.27), studies with publication years over 2010 delivered a significant improvement in the effect of TCM (OR = 1.18, CI: 1.06 to 1.32). Among the total sample size item, there was greater improvement in the group with a relatively larger sample size (*N* > 100) (OR = 1.19, CI: 1.05 to 1.34) than the group with a smaller sample size (*N* ≤ 100) (OR = 1.11, CI: 0.98 to 1.26). In addition, the subgroup that dealt with region diversity revealed that trials conducted in areas with relatively lower income (OR = 1.18, CI: 1.06 to 1.32) proved better improvement compared to that conducted in high-income areas (OR = 1.11, CI: 0.97 to 1.27). All of the above results of the subgroup items may attribute to the design of studies, demographic characteristics of the participants, time line, level of the local economic development, and measurement problems of the research or the fidelity of execution among other issues.

An extensive search strategy was performed based on numerous databases to identify the optimal RCT regarding TS patients treated with TCM. Therefore, the number of included studies and their total sample size contributed directly to complete and robust results. Furthermore, the comprehensive outcomes in our study reliably presented the efficacy and safety of TCM with an approach of quantitative analysis.

Quite a few uncontrollable limitations might jeopardize the conclusion of our results. First, due to the application of TCM, all 47 trials were from China suggesting no original data from other regions in these studies. Moreover, all the included trials have not been registered in the relevant trial platform, which may directly affect the stability of our evidence. The second limitation lied in the quality of the 47 studies included in that 5 [36, 37, 39, 70, 71] and 6 [38, 47, 60, 61, 66, 69] trials were rated as high risk of bias in incomplete outcome data and blinding of outcome assessment, respectively. Thirdly, although we implemented a quantitative analysis to ensure the rationality of different traditional Chinese medicine, heterogeneities ranging from mild to moderate were observed and special attention should be paid to the homogeneity question between them. Finally, the presence of uncontrollable and uncertain biases, including selective reporting biases, unclear detection biases, and other biases (e.g., there existing mild to high heterogeneity between different interventions), is usually inevitable, which may be introduced in our primary analysis and potentially influence the quality of our results.

## 5. Conclusion

In a nutshell, we cautiously suggest that TCM may realize improvement for patients with TS in mitigating the symptoms and its safety may prove itself more acceptable than WM. More high-quality relevant RCTs need to be implemented for the establishment of a comprehensive trial basis in order that more evidence-based researches can be completed successfully.

## Figures and Tables

**Table 1:** Demographic characteristics of literatures.

Publication	Sample size		Proportion of male (%)	Age mean ± SD/range		Diagnose	Therapeutic course	Outcome	ROB
	TCM	CG		TCM	CG				
Wenzhong Zhang, 2013	30	20	86.00%	6-14		CCMD-III	6Ws	①⑤	U
Weibin Gao, 2007	60	60	71.66%	4-21		CCMD-2-R	3Ws	①②③④	U
Dahua Wu, 2007	22	22	75.00%	9.66 ± 2.83	10.03 ± 3.08	DSM-IV	4Ws	①②⑥	L
Guolan Ge, 2013	31	31	79.03%	6.08 ± 2.05		DSM-IV	12Ws	①	H
Linghua Deng, 2014	30	30	70.00%	8.09 ± 1.74	8.47 ± 1.78	DSM-IV	8Ws	①②⑥	H
Congling Sun, 2008	33	33	78.79%	6-18		DSM-IV	24Ws	①③④⑥	L
Anyuan Li, 2008	60	60	71.67%	3-25		DSM-IV	24Ws	①③④⑦	L
Ruiping Ma, 2006	60	60	71.67%	10.26 ± 4.05	10.98 ± 3.80	DSM-IV	24Ws	①③④⑦	L
Anyuan Li, 2013	42	43	70.59%	10.48 ± 2.85	10.53 ± 2.78	DSM-IV	24Ws	①②③④⑥⑦	L
Riming Wu, 2004	30	30	78.33%	4-12		CCMD-2-R	8Ws	①⑦	U
Ying Tang, 2015	50	50	51.00%	8.60 ± 3.20	8.30 ± 3.40	Other	12Ws	①⑦	U
Hengping Chen, 2009	43	30	27.40%	9.20 ± 0.80	9.10 ± 0.90	CCMD-2-R	12Ws	①⑦	U
Guiping Li, 2013	54	54	53.70%	6.50 ± 3.30	7.20 ± 3.80	CCMD-2-R	8Ws	①②	U
Xiubo Du, 2011	32	30	67.74%	7.92 ± 3.59	8.09 ± 3.43	DSM-IV	12Ws	①⑦	U
Yan Liu, 2009	60	40	58.00%	10.26 ± 4.05	10.98 ± 3.80	DSM-IV	24Ws	①③④⑦	U
Bo wang, 2013	48	48	71.88%	13.40 ± 4.20	12.90 ± 3.90	DSM-IV	12Ws	①②	U
Lijun Deng, 2015	40	40	76.25%	9.14 ± 2.21	9.16 ± 2.19	DSM-IV	12Ws	①②⑥	L
Feifei Chen, 2011	30	30	56.67%	8.00 ± 2.36	9.00 ± 2.65	DSM-IV	12Ws	①②	L
Tingting Fu, 2007	33	33	71.21%	7.97 ± 2.79	8.29 ± 2.73	DSM-IV	24Ws	①②③④⑥⑦	L
Feng Han, 2015	30	30	75.00%	8.94 ± 2.18	9.15 ± 2.27	DSM-IV-R	8Ws	①⑥	U
Huawei Li, 2011	36	36	45.83%	9.55 ± 2.91	9.20 ± 3.02	CCMD-III	8Ws	①②⑥	U
Rongyi Zhou, 2016	60	60	78.33%	5-15	5-16	Other	16Ws	①⑦	H
Siyuan Hu, 2014	328	110	80.37%	9.86 ± 2.92	9.35 ± 2.94	Other	4Ws	①②⑥⑦	L
Qi Sun, 2016	36	36	56.00%	4-35		Other	8Ws	①⑦	H
Lifeng Shi, 2009	30	30	70.00%	5-18		DSM-IV	8Ws	①⑦	H
Yi Zheng,2016	362	116	84.52%	9.60 ± 3.10	9.90 ± 2.80	DSM-IV	8Ws	①⑥⑦	L
Jinhui Li, 2016	118	115	54.35%	4-14		DSM-IV	6Ws	①②⑥	L
Xingyou Zhao, 2007	30	30	53.33%	4-18		DSM-IV	12Ws	①②③④⑥⑦	L
Na Yang, 2016	353	118	81.85%	NR		CCMD-III	6Ws	①②⑥⑦	L
Rong Ma, 2010	336	113	NR	4-18		DSM-IV	6Ws	①②③④⑦	H
Zheng Hong, 2015	43	42	84.71%	7.84 ± 1.79	8.31 ± 1.98	DSM-IV-R	12Ws	①②⑤⑥⑦	U
Meiying Liu, 2011	30	30	75.00%	8.17 ± 2.87	9.20 ± 3.16	DSM-IV-TR	8Ws	①②③④⑤⑥⑦	L
Jin Li, 2010	106	105	NR	4-18		CCMD-III	4Ws	①②③④⑤⑥⑦	L
Haiying Wei, 2013	30	30	78.33%	8.53 ± 2.49	8.33 ± 2.48	CCMD-III	8Ws	①②⑥⑦	U
Di Zhang, 2013	30	30	73.33%	NR		CCMD-III	6Ws	①②⑥⑦	U
Chuang Zhao, 2014	30	30	70.00%	8.57 ± 2.52	8.23 ± 2.45	Other	8Ws	①③④	U
L Zhao, 2010	33	31	89.06%	11.95 ± 2.93	12.50 ± 2.87	DSM-IV-TR	8Ws	①②③④	L
Anyuan Li, 2009	60	30	57.78%	9.59 ± 3.00	9.60 ± 2.95	DSM-IV	24Ws	①②③④⑤⑦	L
Min Wu, 2010	31	30	83.61%	8.61 ± 3.16	9.30 ± 2.32	ICD-10	24Ws	①②	L
Min Wu, 2009	41	40	81.48%	9.70 ± 2.01	9.10 ± 1.13	DSM-IV	24Ws	①②	L
Yunchou Wu,2015	32	32	70.31%	5-18		DSM-IV	6Ws	①⑥⑦	U
Feng Yang, 2012	30	30	61.67%	7.5	7.3	DSM-IV	4Ws	①⑤	H
Fen Wang, 2011	30	30	76.67%	4-12		Other	4Ws	①⑦	U
Xinhui Shan, 2016	45	45	81.11%	8.00 ± 2.5	9.00 ± 2.7	Other	12Ws	①②⑤⑦	U
Jiaomei Feng, 2011	33	33	71.21%	8.48 ± 2.50	8.18 ± 2.57	DSM-IV	12Ws	①②③④⑤⑥⑦	U
Jinping Fan, 2017	60	60	64.17%	10.33 ± 3.14	10.90 ± 3.54	DSM-V	12Ws	①②⑥⑦	U
Jingyu Qiu, 2010	60	40	58.00%	11.03 ± 4.05	11.09 ± 3.80	DSM-IV	24Ws	①③④⑦	U

CCMD: Chinese Classification of Mental Disorders; CG: control group; DSM: the Diagnostic and Statistical Manual; H: high; L: Low; U: unclear; NR: not reported; ROB: risk of bias; TCM: traditional Chinese medicine; outcome: ① clinical efficacy; ② YGTSS (Yale Global Tic Severity Scale); ③ YGTSS motor tic scores; ④ YGTSS vocal tic scores; ⑤ recurrence rate; ⑥ TCMSSS (TCM Syndrome Score Scale); ⑦ adverse reaction.

**Table 2:** Primary results based on various outcomes and subgroup analyses.

Meta-analyses variables	No. of studies	No. of patients		Pool effect size	*I* ^2^
		TCM	CG		
*Numerical variable*				*Pooled ORs (95% CI)*	
Clinical efficacy	47	3231	2206	1.15 (1.06 to 1.26)	0.00%
YGTSS motor tic scores	9	396	356	1.26 (1.00 to 1.58)	0.00%
YGTSS vocal tic scores	9	396	356	1.17 (0.92 to 1.49)	0.00%
Recurrence rate	6	241	200	0.44 (0.24 to 0.78)	0.00%
TCMSSS	14	1265	807	1.20 (1.04 to 1.37)	0.00%
Adverse reaction	29	2504	1496	0.32 (0.24 to 0.43)	32.90%
*Continuous variable*				*Pooled SMDs (95% CI)*	
YGTSS total scores	26	2401	1441	-0.21 (-0.29 to -0.14)	12.30%
YGTSS motor tic scores	7	670	415	-0.37 (-0.55 to -0.19)	41.50%
YGTSS vocal tic scores	7	670	415	-0.23 (-0.35 to -0.10)	0.00%
TCMSSS	6	194	195	-0.69 (-1.17 to -0.21)	80.50%
*Subgroup analysis based on the outcome of YGTSS*				*Pooled SMDs (95% CI)*	
*Disease course*					
Overall	33	1653	1308	1.14 (1.02 to 1.28)	0.00%
Average course≤3Ys	24	1198	936	1.14 (0.99 to 1.30)	0.00%
Average course>3Ys	9	455	372	1.15 (0.93 to 1.42)	0.00%
*Condition of disease*					
Overall	9	299	268	1.08 (0.84 to 1.38)	0.00%
Mild	3	121	90	1.06 (0.71 to 1.61)	0.00%
Moderate-severe	6	178	178	1.09 (0.80 to 1.48)	0.00%
*Publication year*					
Overall	47	3231	2206	1.15 (1.06 to 1.26)	0.00%
≤2010	18	1128	817	1.10 (0.96 to 1.27)	0.00%
>2010	29	2103	1389	1.18 (1.06 to 1.32)	0.00%
*Therapeutic course*					
Overall	47	3231	2206	1.15 (1.06 to 1.26)	0.00%
≤12Ws	24	2206	1268	1.18 (1.05 to 1.32)	0.00%
>12Ws	23	1025	938	1.12 (0.96 to 1.28)	0.00%
*Diagnostic standards*					
Overall	47	3231	2206	1.15 (1.06 to 1.26)	0.00%
CCMD	11	1100	623	1.18 (1.00 to 1.39)	4.30%
DSM	29	1849	1302	1.15 (1.09 to 1.29)	0.00%
Other	7	282	281	1.10 (0.85 to 1.41)	0.00%
*Total sample size*					
Overall	47	3231	2206	1.15 (1.06 to 1.26)	0.00%
≤100	32	1104	1045	1.11 (0.98 to 1.26)	0.00%
>100	15	2127	1161	1.19 (1.05 to 1.34)	6.30%
*Region*					
Overall	47	3231	2206	1.15 (1.06 to 1.26)	0.00%
High income	26	2078	1053	1.11 (0.97 to 1.27)	0.00%
Low and middle income	21	1153	1153	1.18 (1.06 to 1.32)	0.00%
*ROB quality*					
Overall	47	3231	2206	1.15 (1.06 to 1.26)	0.00%
High	7	989	945	1.15 (1.06 to 1.26)	0.00%
Low/unclear	40	2242	1261	1.16 (1.06 to 1.28)	0.00%

CI: confidence interval; CCMD: Chinese Classification of Mental Disorders; CG: control group; DSM: the Diagnostic and Statistical Manual; OR: odds ratio; ROB: risk of bias; SMD: standard mean differences; TCM: traditional Chinese medicine; TCMSSS: TCM Syndrome Score Scale; YGTSS: Yale Global Tic Severity Scale.

## Supplementary Materials

Supplementary 1 PubMed strategy.

Supplementary 2 Supplement Figure 1: risk of bias graph.

Supplementary 3 Supplement Figure 2: risk of bias summary.

Supplementary 4 Supplement Figure 3: funnel plots of clinical efficacy.

Supplementary 5 Supplement Figure 4: Egger plot of clinical efficacy.
