# Supplementary material for: Traditional Chinese Medicine Strategy for Patients with Tourette Syndrome Based on Clinical Efficacy and Safety: A Meta-Analysis of 47 Randomized Controlled Trials
Source: Biomed Res Int. 2021 Mar 10;2021:6630598. doi: 10.1155/2021/6630598 (PMC7977981; doi:10.1155/2021/6630598)
Supplement: Supplementary 1 — PubMed strategy. [file 6630598.f1.docx]

**Pubmed strategy**

1. "Tourette Syndrome"[Mesh]
2. Syndrome, Tourette[Title/Abstract]) OR (Combined Vocal[Title/Abstract] AND Syndrome[Title/Abstract])) OR (Gilles de la Tourette's Disease[Title/Abstract])) OR (Multiple Motor[Title/Abstract] AND Vocal Tic Disorder, Combined[Title/Abstract])) OR (Gilles De La Tourette's Syndrome[Title/Abstract])) OR (Tourette Disease[Title/Abstract])) OR (Tourette Disorder[Title/Abstract])) OR (Tourette's Disease[Title/Abstract])) OR (Tourettes Disease[Title/Abstract])) OR (Tourette's Disorder[Title/Abstract])) OR (Tourettes Disorder[Title/Abstract])) OR (Tourette's Syndrome[Title/Abstract])) OR (Tourettes Syndrome[Title/Abstract])) OR (Chronic Motor[Title/Abstract] AND Vocal Tic Disorder[Title/Abstract])) OR (Combined Multiple Motor[Title/Abstract] AND Vocal Tic Disorder[Title/Abstract])) OR (Tic Disorder, Combined Vocal[Title/Abstract] AND Multiple Motor[Title/Abstract])
3. "Medicine, Chinese Traditional"[Mesh]
4. (Traditional Chinese Medicine[Title/Abstract]) OR (Chung I Hsueh[Title/Abstract])) OR (Hsueh, Chung I[Title/Abstract])) OR (Traditional Medicine, Chinese[Title/Abstract])) OR (Zhong Yi Xue[Title/Abstract])) OR (Chinese Traditional Medicine[Title/Abstract])) OR (Chinese Medicine, Traditional[Title/Abstract])) OR (Traditional Tongue Diagnosis[Title/Abstract])) OR (Tongue Diagnoses, Traditional[Title/Abstract])) OR (Tongue Diagnosis, Traditional[Title/Abstract])) OR (Traditional Tongue Diagnoses[Title/Abstract])) OR (Traditional Tongue Assessment[Title/Abstract])) OR (Tongue Assessment, Traditional[Title/Abstract])) OR (Traditional Tongue Assessments[Title/Abstract])
5. "Drugs, Chinese Herbal"[MeSH Terms]
6. (((((Chinese Drugs, Plant[Title/Abstract]) OR (Chinese Herbal Drugs[Title/Abstract])) OR (Herbal Drugs, Chinese[Title/Abstract])) OR (Plant Extracts, Chinese[Title/Abstract])) OR (Chinese Plant Extracts[Title/Abstract])) OR (Extracts, Chinese Plant[Title/Abstract])
7. randomized controlled trial[Publication Type] OR randomized[Title/Abstract] OR placebo[Title/Abstract]OR RCT[Title/Abstract]OR rct[Title/Abstract]
8. ("Tourette Syndrome"[Mesh]) OR (((((((((((((((((Syndrome, Tourette[Title/Abstract]) OR (Combined Vocal[Title/Abstract] AND Multiple Motor Tic Disorder[Title/Abstract])) OR (Gilles de la Tourette Syndrome[Title/Abstract])) OR (Gilles de la Tourette's Disease[Title/Abstract])) OR (Multiple Motor[Title/Abstract] AND Vocal Tic Disorder, Combined[Title/Abstract])) OR (Gilles De La Tourette's Syndrome[Title/Abstract])) OR (Tourette Disease[Title/Abstract])) OR (Tourette Disorder[Title/Abstract])) OR (Tourette's Disease[Title/Abstract])) OR (Tourettes Disease[Title/Abstract])) OR (Tourette's Disorder[Title/Abstract])) OR (Tourettes Disorder[Title/Abstract])) OR (Tourette's Syndrome[Title/Abstract])) OR (Tourettes Syndrome[Title/Abstract])) OR (Chronic Motor[Title/Abstract] AND Vocal Tic Disorder[Title/Abstract])) OR (Combined Multiple Motor[Title/Abstract] AND Vocal Tic Disorder[Title/Abstract])) OR (Tic Disorder, Combined Vocal[Title/Abstract] AND Multiple Motor[Title/Abstract]))
9. ((((("Medicine, Chinese Traditional"[Mesh]) OR ((((((((((((((Traditional Chinese Medicine[Title/Abstract]) OR (Chung I Hsueh[Title/Abstract])) OR (Hsueh, Chung I[Title/Abstract])) OR (Traditional Medicine, Chinese[Title/Abstract])) OR (Zhong Yi Xue[Title/Abstract])) OR (Chinese Traditional Medicine[Title/Abstract])) OR (Chinese Medicine, Traditional[Title/Abstract])) OR (Traditional Tongue Diagnosis[Title/Abstract])) OR (Tongue Diagnoses, Traditional[Title/Abstract])) OR (Tongue Diagnosis, Traditional[Title/Abstract])) OR (Traditional Tongue Diagnoses[Title/Abstract])) OR (Traditional Tongue Assessment[Title/Abstract])) OR (Tongue Assessment, Traditional[Title/Abstract])) OR (Traditional Tongue Assessments[Title/Abstract]))) OR ("Drugs, Chinese Herbal"[MeSH Terms])) OR ((((((Chinese Drugs, Plant[Title/Abstract]) OR (Chinese Herbal Drugs[Title/Abstract])) OR (Herbal Drugs, Chinese[Title/Abstract])) OR (Plant Extracts, Chinese[Title/Abstract])) OR (Chinese Plant Extracts[Title/Abstract])) OR (Extracts, Chinese Plant[Title/Abstract])))
10. ((("Tourette Syndrome"[Mesh]) OR (((((((((((((((((Syndrome, Tourette[Title/Abstract]) OR (Combined Vocal[Title/Abstract] AND Multiple Motor Tic Disorder[Title/Abstract])) OR (Gilles de la Tourette Syndrome[Title/Abstract])) OR (Gilles de la Tourette's Disease[Title/Abstract])) OR (Multiple Motor[Title/Abstract] AND Vocal Tic Disorder, Combined[Title/Abstract])) OR (Gilles De La Tourette's Syndrome[Title/Abstract])) OR (Tourette Disease[Title/Abstract])) OR (Tourette Disorder[Title/Abstract])) OR (Tourette's Disease[Title/Abstract])) OR (Tourettes Disease[Title/Abstract])) OR (Tourette's Disorder[Title/Abstract])) OR (Tourettes Disorder[Title/Abstract])) OR (Tourette's Syndrome[Title/Abstract])) OR (Tourettes Syndrome[Title/Abstract])) OR (Chronic Motor[Title/Abstract] AND Vocal Tic Disorder[Title/Abstract])) OR (Combined Multiple Motor[Title/Abstract] AND Vocal Tic Disorder[Title/Abstract])) OR (Tic Disorder, Combined Vocal[Title/Abstract] AND Multiple Motor[Title/Abstract]))) AND (((((("Medicine, Chinese Traditional"[Mesh]) OR ((((((((((((((Traditional Chinese Medicine[Title/Abstract]) OR (Chung I Hsueh[Title/Abstract])) OR (Hsueh, Chung I[Title/Abstract])) OR (Traditional Medicine, Chinese[Title/Abstract])) OR (Zhong Yi Xue[Title/Abstract])) OR (Chinese Traditional Medicine[Title/Abstract])) OR (Chinese Medicine, Traditional[Title/Abstract])) OR (Traditional Tongue Diagnosis[Title/Abstract])) OR (Tongue Diagnoses, Traditional[Title/Abstract])) OR (Tongue Diagnosis, Traditional[Title/Abstract])) OR (Traditional Tongue Diagnoses[Title/Abstract])) OR (Traditional Tongue Assessment[Title/Abstract])) OR (Tongue Assessment, Traditional[Title/Abstract])) OR (Traditional Tongue Assessments[Title/Abstract]))) OR ("Drugs, Chinese Herbal"[MeSH Terms])) OR ((((((Chinese Drugs, Plant[Title/Abstract]) OR (Chinese Herbal Drugs[Title/Abstract])) OR (Herbal Drugs, Chinese[Title/Abstract])) OR (Plant Extracts, Chinese[Title/Abstract])) OR (Chinese Plant Extracts[Title/Abstract])) OR (Extracts, Chinese Plant[Title/Abstract]))) AND (randomized controlled trial[Publication Type] OR randomized[Title/Abstract] OR placebo[Title/Abstract]OR RCT[Title/Abstract]OR rct[Title/Abstract])

**Embase strategy**

1. 'gilles de la tourette syndrome'/exp
2. 'syndrome, tourette':ab,ti OR 'combined vocal and multiple motor tic disorder':ab,ti OR 'gilles de la tourette syndrome':ab,ti OR 'gilles de la tourettes disease':ab,ti OR 'multiple motor and vocal tic disorder, combined':ab,ti OR 'gilles de la tourettes syndrome':ab,ti OR 'tourette disease':ab,ti OR 'tourettes disease':ab,ti OR 'tourette s disorder':ab,ti OR 'tourettes disorder':ab,ti OR 'tourettes syndrome':ab,ti OR 'chronic motor and vocal tic disorder':ab,ti OR 'combined multiple motor and vocal tic disorder':ab,ti OR 'tic disorder, combined vocal and multiple motor':ab,ti
3. 'chinese medicine'/exp OR 'herbaceous agent'/exp
4. 'traditional medicine, chinese':ab,ti OR 'zhong yi xue':ab,ti OR 'chinese traditional medicine':ab,ti OR 'chinese medicine, traditional':ab,ti OR 'traditional tongue diagnosis':ab,ti OR 'tongue diagnoses, traditional':ab,ti OR 'tongue diagnosis, traditional':ab,ti OR 'traditional tongue diagnoses':ab,ti OR 'traditional tongue assessment':ab,ti OR 'tongue assessment, traditional':ab,ti OR 'traditional chinese medicine':ab,ti OR 'chung i hsueh':ab,ti OR 'hsueh, chung i':ab,ti OR 'traditional tongue assessments':ab,ti
5. 'chinese drugs, plant':ab,ti OR 'chinese herbal drugs':ab,ti OR 'herbal drugs, chinese':ab,ti OR 'plant extracts, chinese':ab,ti OR 'chinese plant extracts':ab,ti OR 'extracts, chinese plant':ab,ti
6. 'random':ab,ti OR 'double-blind':ab,ti OR 'randomized controlled trial':ab,ti OR 'randomized':ab,ti OR 'placebo':ab,ti OR 'rct':ab,ti
7. #1 OR #2
8. #3 OR #4 OR #5
9. #6 AND #7 AND #8

**Cochrane library search strategy**

1. MeSH descriptor: [Tourette Syndrome] explode all trees
2. (Syndrome, Tourette):ti,ab,kw OR (Combined Vocal and Multiple Motor Tic Disorder):ti,ab,kw OR (Gilles de la Tourette Syndrome):ti,ab,kw OR (Gilles de la Tourette's Disease):ti,ab,kw OR (Multiple Motor and Vocal Tic Disorder, Combined):ti,ab,kw OR (Gilles De La Tourette's Syndrome):ti,ab,kw OR (Tourette Disease):ti,ab,kw OR (Tourette Disorder):ti,ab,kw OR (Tourette's Disease):ti,ab,kw OR (Tourettes Disease):ti,ab,kw OR (Tourette's Disorder):ti,ab,kw OR (Tourettes Disorder):ti,ab,kw OR (Tourette's Syndrome):ti,ab,kw OR (Tourettes Syndrome):ti,ab,kw OR (Chronic Motor and Vocal Tic Disorder):ti,ab,kw OR (Combined Multiple Motor and Vocal Tic Disorder):ti,ab,kw OR (Tic Disorder, Combined Vocal and Multiple Motor):ti,ab,kw
3. MeSH descriptor: [Medicine, Chinese Traditional] explode all trees
4. (Traditional Chinese Medicine):ti,ab,kw OR (Chung I Hsueh):ti,ab,kw OR (Hsueh, Chung I):ti,ab,kw OR (Traditional Medicine, Chinese):ti,ab,kw OR (Zhong Yi Xue):ti,ab,kw OR (Chinese Traditional Medicine):ti,ab,kw OR (Chinese Medicine, Traditional):ti,ab,kw OR (Traditional Tongue Diagnosis):ti,ab,kw OR (Tongue Diagnoses, Traditional):ti,ab,kw OR (Tongue Diagnosis, Traditional):ti,ab,kw OR (Traditional Tongue Diagnoses):ti,ab,kw OR (Traditional Tongue Assessment):ti,ab,kw OR (Tongue Assessment, Traditional):ti,ab,kw OR (Traditional Tongue Assessments):ti,ab,kw
5. MeSH descriptor: [Drugs, Chinese Herbal] explode all trees
6. (Chinese Drugs, Plant):ti,ab,kw OR (Chinese Herbal Drugs):ti,ab,kw OR (Herbal Drugs, Chinese):ti,ab,kw OR (Plant Extracts, Chinese):ti,ab,kw OR (Chinese Plant Extracts):ti,ab,kw OR (Extracts, Chinese Plant):ti,ab,kw
7. #1 OR #2
8. #3 OR #4 OR #5 OR #6
9. #7 AND #8

**Web of science search strategy**

1. TS=(tourette syndrome OR Syndrome, Tourette OR Combined Vocal and Multiple Motor Tic Disorder OR Gilles de la Tourette Syndrome OR Gilles de la Tourette's Disease OR Multiple Motor and Vocal Tic Disorder, Combined OR Gilles De La Tourette's Syndrome OR Tourette Disease OR Tourette Disorder OR Tourette's Disease OR Tourettes Disease OR Tourette's Disorder OR Tourettes Disorder OR Tourette's Syndrome OR Tourettes Syndrome OR Chronic Motor and Vocal Tic Disorder OR Combined Multiple Motor and Vocal Tic Disorder OR Tic Disorder, Combined Vocal and Multiple Motor) 数据库= WOS, KJD, MEDLINE, RSCI, SCIELO 时间跨度=所有年份检索语言=自动
2. TS=(Medicine, Chinese Traditional OR Traditional Chinese Medicine OR Chung I Hsueh OR Hsueh, Chung I OR Traditional Medicine, Chinese OR Zhong Yi Xue OR Chinese Traditional Medicine OR Chinese Medicine, Traditional OR Traditional Tongue Diagnosis OR Tongue Diagnoses, Traditional OR Tongue Diagnosis, Traditional OR Traditional Tongue Diagnoses OR Traditional Tongue Assessment OR Tongue Assessment, Traditional OR Traditional Tongue Assessments OR Drugs, Chinese Herbal OR Chinese Drugs, Plant OR Chinese Herbal Drugs OR Herbal Drugs, Chinese OR Plant Extracts, Chinese OR Chinese Plant Extracts OR Extracts, Chinese Plant OR Acupuncture OR Pharmacopuncture OR Acupuncture Therapy OR Acupuncture Treatment OR Acupuncture Treatments OR Treatment, Acupuncture OR Therapy, Acupuncture OR Pharmacoacupuncture Treatment OR Treatment, Pharmacoacupuncture OR Pharmacoacupuncture Therapy OR Therapy, Pharmacoacupuncture OR Acupotomy OR Acupotomies) 数据库= WOS, KJD, MEDLINE, RSCI, SCIELO 时间跨度=所有年份检索语言=自动
3. TS=(randomized controlled trial OR randomized OR placebo OR RCT OR rct) 数据库= WOS, KJD, MEDLINE, RSCI, SCIELO 时间跨度=所有年份检索语言=自动
4. #3 AND #2 AND #1 数据库= WOS, KJD, MEDLINE, RSCI, SCIELO 时间跨度=所有年份检索语言=自动

**CBM search strategy**

1. "抽动秽语综合征"[常用字段:智能] OR "gilles"[常用字段:智能] AND "de"[常用字段:智能] AND "la"[常用字段:智能] AND "tourette"[常用字段:智能] AND "syndrome"[常用字段:智能] OR "tourette"[常用字段:智能] AND "syndrome"[常用字段:智能] OR "多发性抽动症"[常用字段:智能]
2. "中医学"[不加权:扩展]
3. "中医药"[常用字段:智能] OR "中药"[常用字段:智能]
4. "随机对照试验"[不加权:扩展]
5. "随机分组"[常用字段:智能] OR "随机"[常用字段:智能] OR "RCT"[常用字段:智能] OR "rct"[常用字段:智能]
6. "中医学"[不加权:扩展] OR "中医药"[常用字段:智能] OR "中药"[常用字段:智能]
7. "随机对照试验"[不加权:扩展] OR "随机分组"[常用字段:智能] OR "随机"[常用字段:智能] OR "RCT"[常用字段:智能] OR "rct"[常用字段:智能]
8. "抽动秽语综合征"[常用字段:智能] OR "gilles"[常用字段:智能] AND "de"[常用字段:智能] AND "la"[常用字段:智能] AND "tourette"[常用字段:智能] AND "syndrome"[常用字段:智能] OR "tourette"[常用字段:智能] AND "syndrome"[常用字段:智能] OR "多发性抽动症"[常用字段:智能] AND "中医学"[不加权:扩展] OR "中医药"[常用字段:智能] OR "中药"[常用字段:智能] AND "随机对照试验"[不加权:扩展] OR "随机分组"[常用字段:智能] OR "随机"[常用字段:智能] OR "RCT"[常用字段:智能] OR "rct"[常用字段:智能]

**VIP search strategy**

1. 题名或关键词=抽动秽语综合征 OR 题名或关键词=gilles de la tourette syndrome) OR 题名或关键词=tourette syndrome) OR 题名或关键词=多发性抽动症)

AND

1. ((((((((题名或关键词=中医 OR 题名或关键词=中医药) OR 题名或关键词=中药)

AND

1. ((((文摘=随机对照 OR 文摘=随机分组) OR文摘=随机) OR 文摘=rct) OR 文摘=RCT)))))

**CNKI search strategy**

1. (主题=中英文扩展(抽动秽语综合征,中英文对照) ) 或者 ( 题名=中英文扩展(抽动秽语综合征,中英文对照) ) 或者 ( v_subject=中英文扩展(抽动秽语综合征,中英文对照) ) 或者 ( title=中英文扩展(抽动秽语综合征,中英文对照) )) 或者 (( 主题=中英文扩展(gilles de la tourette syndrome,中英文对照) ) 或者 ( 题名=中英文扩展(gilles de la tourette syndrome,中英文对照) ) 或者 ( v_subject=中英文扩展(gilles de la tourette syndrome,中英文对照) ) 或者 ( title=中英文扩展(gilles de la tourette syndrome,中英文对照) )) ) 或者 ( (( 主题=中英文扩展(tourette syndrome,中英文对照) ) 或者 ( 题名=中英文扩展(tourette syndrome,中英文对照) ) 或者 ( v_subject=中英文扩展(tourette syndrome,中英文对照) ) 或者 ( title=中英文扩展(tourette syndrome,中英文对照) )) 或者 (( 主题=中英文扩展(多发性抽动症,中英文对照) ) 或者 ( 题名=中英文扩展(多发性抽动症,中英文对照) ) 或者 ( v_subject=中英文扩展(多发性抽动症,中英文对照) ) 或者 ( title=中英文扩展(多发性抽动症,中英文对照)

**AND**

1. 主题=中英文扩展(中医,中英文对照) ) 或者 ( 题名=中英文扩展(中医,中英文对照) ) 或者 ( v_subject=中英文扩展(中医,中英文对照) ) 或者 ( title=中英文扩展(中医,中英文对照) )) 或者 (( 主题=中英文扩展(中医药,中英文对照) ) 或者 ( 题名=中英文扩展(中医药,中英文对照) ) 或者 ( v_subject=中英文扩展(中医药,中英文对照) ) 或者 ( title=中英文扩展(中医药,中英文对照) )) ) 或者 ( (( 主题=中英文扩展(中药,中英文对照) ) 或者 ( 题名=中英文扩展(中药,中英文对照) ) 或者 ( v_subject=中英文扩展(中药,中英文对照) ) 或者 ( title=中英文扩展(中药,中英文对照) ))

**AND**

1. (主题=中英文扩展(随机对照,中英文对照) ) 或者 ( 题名=中英文扩展(随机对照,中英文对照) ) 或者 ( v_subject=中英文扩展(随机对照,中英文对照) ) 或者 ( title=中英文扩展(随机对照,中英文对照) )) 或者 (( 主题=中英文扩展(随机分组,中英文对照) ) 或者 ( 题名=中英文扩展(随机分组,中英文对照) ) 或者 ( v_subject=中英文扩展(随机分组,中英文对照) ) 或者 ( title=中英文扩展(随机分组,中英文对照) )) ) 或者 ( (( 主题=中英文扩展(随机,中英文对照) ) 或者 ( 题名=中英文扩展(随机,中英文对照) ) 或者 ( v_subject=中英文扩展(随机,中英文对照) ) 或者 ( title=中英文扩展(随机,中英文对照) )) 或者 (( 主题=中英文扩展(rct,中英文对照) ) 或者 ( 题名=中英文扩展(rct,中英文对照) ) 或者 ( v_subject=中英文扩展(rct,中英文对照) ) 或者 ( title=中英文扩展(rct,中英文对照) )) ) ) 或者 (( 主题=中英文扩展(RCT,中英文对照) ) 或者 ( 题名=中英文扩展(RCT,中英文对照) ) 或者 ( v_subject=中英文扩展(RCT,中英文对照) ) 或者 ( title=中英文扩展(RCT,中英文对照) (模糊匹配)

**Wanfang data search strategy**

1. (主题:(抽动秽语综合征+gilles de la tourette syndrome+tourette syndrome+多发性抽动症)

*AND

1. 主题:(中医+中医药+中药)

*AND

1. 主题:(随机对照+随机分组+随机+rct+RCT))
