# Supplementary material for: Traditional Chinese Medicine Strategy for Patients with Tourette Syndrome Based on Clinical Efficacy and Safety: A Meta-Analysis of 47 Randomized Controlled Trials
Source: Biomed Res Int. 2021 Mar 10;2021:6630598. doi: 10.1155/2021/6630598 (PMC7977981; doi:10.1155/2021/6630598)
Supplement: Supplementary 2 — Supplement Figure 1: risk of bias graph. [file 6630598.f2.pdf]

Supplement Figure.1 Risk of Bias graph

Items							Author, year
L	U	U	U	L	U	U	Wenzhong Zhang, 2013
L	U	U	U	L	U	U	Weibin Gao, 2007
L	U	L	H	L	U	L	Dahua Wu, 2007
L	U	U	U	H	H	U	Guolan Ge, 2013
L	U	U	U	H	U	U	Linghua Deng, 2014
L	L	U	U	L	L	L	Congling Sun, 2008
L	U	U	U	L	U	L	Anyuan Li, 2008
L	U	U	U	L	U	L	Ruiping Ma, 2006
L	U	U	U	L	U	L	Anyuan Li, 2013
L	U	U	U	L	U	U	Riming Wu, 2004
L	U	U	U	L	U	U	Ying Tang, 2015
L	U	U	U	L	U	U	Hengping Chen, 2009
L	U	U	U	L	U	U	Guiping Li, 2013
L	U	U	U	L	U	U	Xiubo Du, 2011
L	U	U	U	L	U	U	Yan Liu, 2009
L	U	U	U	L	U	U	Bo wang, 2013
L	U	U	U	L	U	L	Lijun Deng, 2015
L	U	U	U	L	U	L	Feifei Chen, 2011
L	L	L	U	L	L	L	Tingting Fu, 2007
L	U	U	U	L	U	U	Feng Han, 2015
L	U	U	U	L	U	U	Huawei Li, 2011

L	U	U	U	L	U	H	Rongyi Zhou, 2016
L	U	U	U	L	U	L	Siyuan Hu, 2014
L	U	U	U	L	U	H	Qi Sun, 2016
L	U	U	U	H	U	U	Lifeng Shi, 2009
L	L	L	H	L	L	L	Yi Zheng,2016
L	U	L	H	L	L	L	Jinhui Li, 2016
L	U	L	L	L	U	L	Xingyou Zhao, 2007
L	U	L	H	L	L	L	Na Yang, 2016
L	U	U	U	H	U	H	Rong Ma, 2010
L	U	U	U	L	U	U	Zheng Hong, 2015
L	U	U	U	L	U	L	Meiying Liu, 2011
L	U	L	H	L	U	L	Jin Li, 2010
L	U	U	U	L	U	U	Haiying Wei, 2013
L	U	U	U	L	U	U	Di Zhang, 2013
L	U	U	U	L	U	U	Chuang Zhao, 2014
L	U	L	H	L	L	L	L Zhao,2010
L	U	U	U	L	U	L	Anyuan Li, 2009
L	U	U	U	L	U	L	Min Wu, 2010
L	U	U	U	L	L	L	Min Wu, 2009
L	U	U	U	L	U	U	Yunchou Wu,2015
L	U	U	U	H	H	U	Feng Yang, 2012
L	U	U	U	L	U	U	Fen Wang, 2011
L	U	U	U	L	U	U	Xinhui Shan, 2016

Random sequence generation (selection bias)	Allocation concealment (selection bias)	Blinding of participants and personnel (performance bias)	Blinding of outcome assessment (detection bias)	Incomplete outcome data (attrition bias)	Selective reporting (reporting bias)	Other bias	
L	U	U	U	L	L	U	Jiaomei Feng, 2011
L	U	U	U	L	U	U	Jinping Fan, 2017
L	U	U	U	L	U	U	Jingyu Qiu, 2010

Low risk

Unclear risk

High risk
