# Supplementary figures and images for: Traditional Chinese Medicine Strategy for Patients with Tourette Syndrome Based on Clinical Efficacy and Safety: A Meta-Analysis of 47 Randomized Controlled Trials
Source: Biomed Res Int. 2021 Mar 10;2021:6630598. doi: 10.1155/2021/6630598 (PMC7977981; doi:10.1155/2021/6630598)

Supplement Figure.2 Risk of Bias Summary

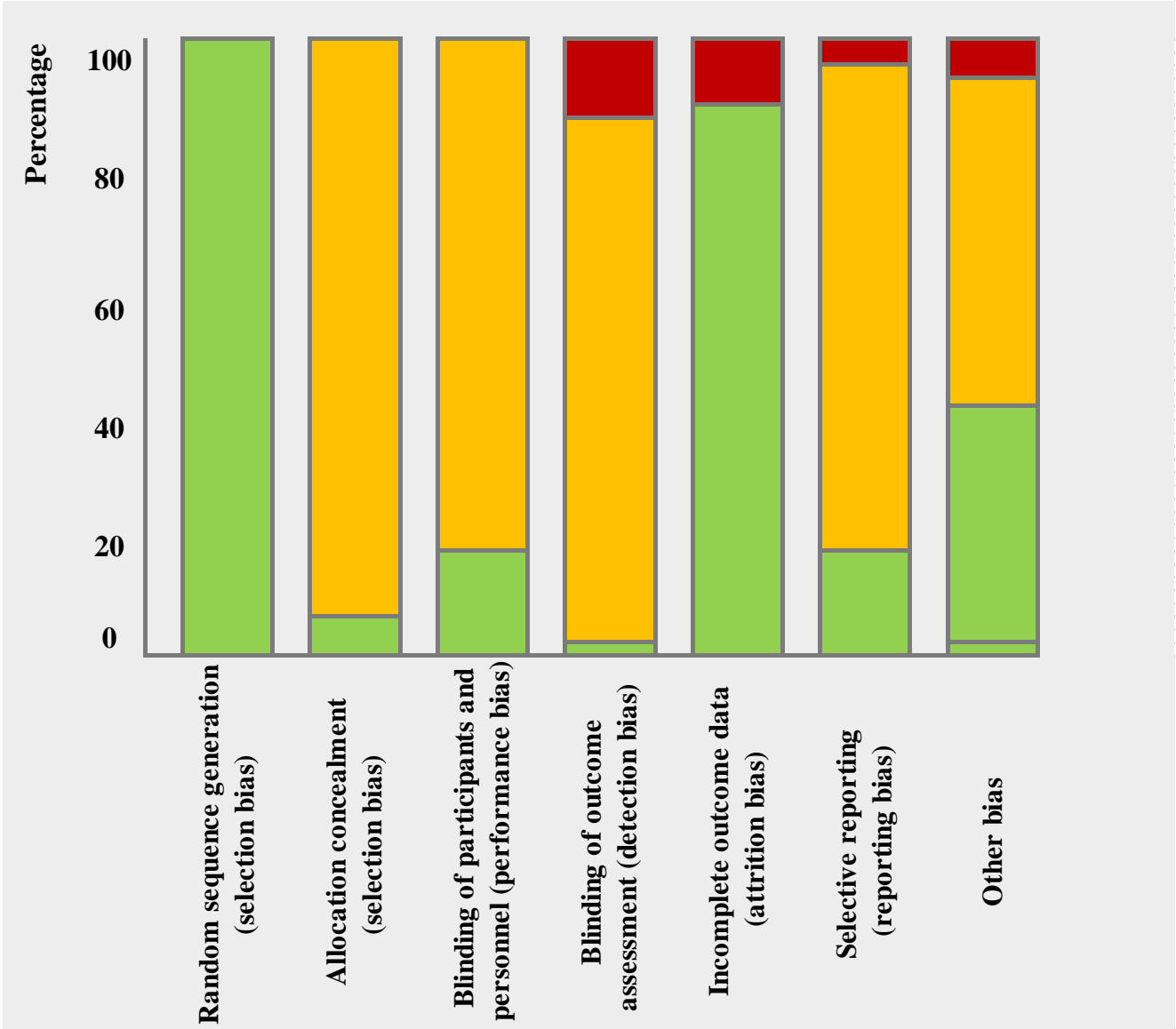

Supplement: Supplementary 3 — Supplement Figure 2: risk of bias summary. [file 6630598.f3.pdf]

Funnel plot with pseudo 95% confidence limits

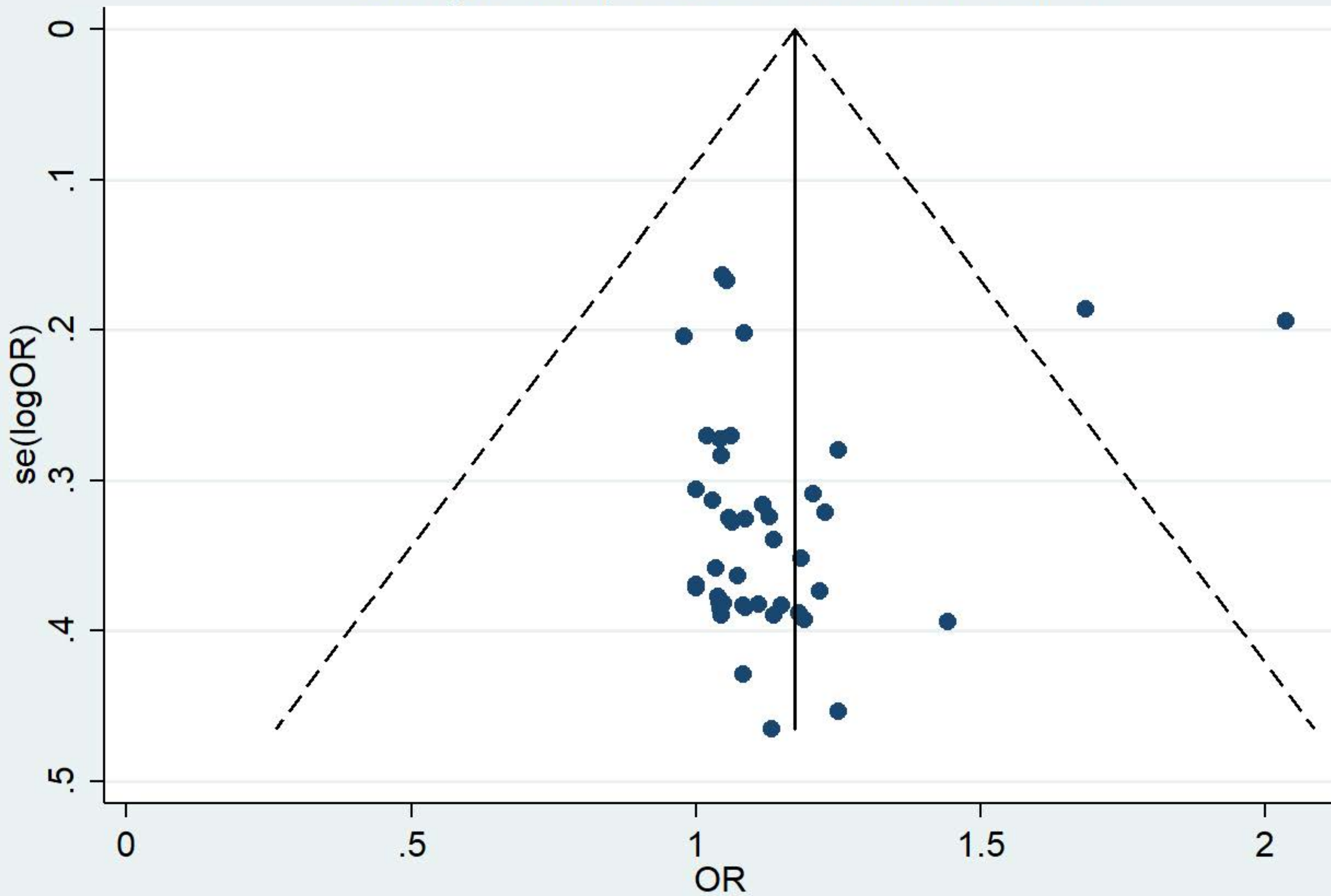

Supplement: Supplementary 4 — Supplement Figure 3: funnel plots of clinical efficacy. [file 6630598.f4.pdf]

Egger's publication bias plot

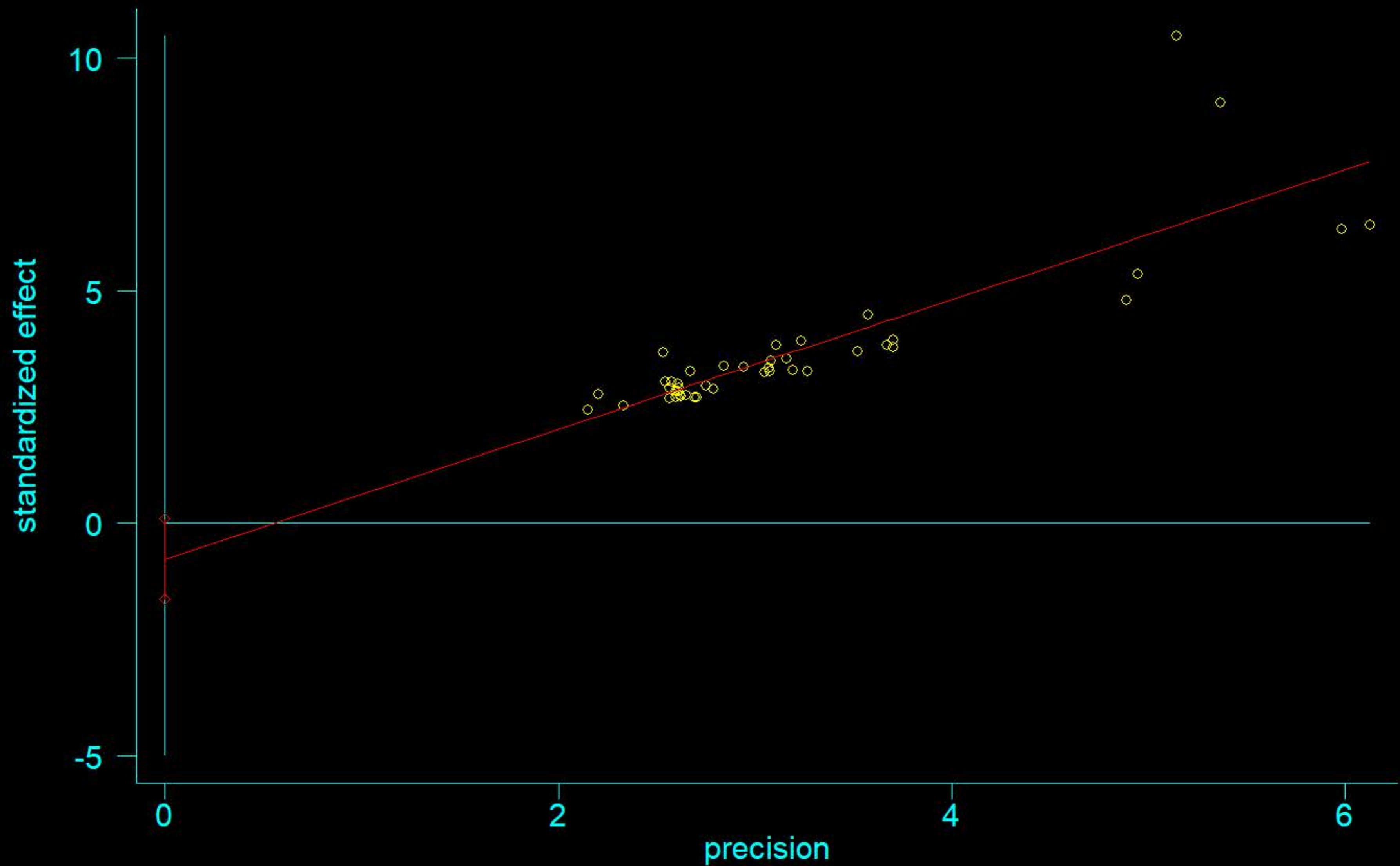

Supplement: Supplementary 5 — Supplement Figure 4: Egger plot of clinical efficacy. [file 6630598.f5.pdf]
